# Characteristics of colonic migrating motor complexes in neuronal NOS (nNOS) knockout mice

**DOI:** 10.3389/fnins.2013.00184

**Published:** 2013-10-14

**Authors:** Nick J. Spencer

**Affiliations:** ^1^Discipline of Human Physiology, Center for Neuroscience, School of Medicine, Flinders UniversityAdelaide, SA, Australia; ^2^Department of Physiology & Cell Biology, University of Nevada School of MedicineReno, NV, USA

**Keywords:** colonic migrating motor complex, nitric oxide, nitric oxide synthase, peristalsis, mouse, cre-lox genetics, compensation

## Abstract

It is well established that the intrinsic pacemaker mechanism that generates cyclical colonic migrating motor complexes (CMMCs) does not require endogenous nitric oxide (NO). However, pharmacological blockade of endogenous NO production potently increases the frequency of CMMCs, suggesting that endogenous NO acts normally to inhibit the CMMC pacemaker mechanism. In this study, we investigated whether mice with a life long genetic deletion of the neuronal nitric oxide synthase (nNOS) gene would show similar CMMC characteristics as wild type mice that have endogenous NO production acutely inhibited. Intracellular electrophysiological and mechanical recordings were made from circular muscle cells of isolated whole mouse colon in wild type and nNOS knockout (KO) mice at 35°C. In wild type mice, the NOS inhibitor, L-NA (100 μM) caused a significant increase in CMMC frequency and a significant depolarization of the CM layer. However, unexpectedly, the frequency of CMMCs in nNOS KO mice was not significantly different from control mice. Also, the resting membrane potential of CM cells in nNOS KO mice was not depolarized compared to controls; and the amplitude of the slow depolarization phase underlying MCs was of similar amplitude between KO and wild type offspring. These findings show that in nNOS KO mice, the major characteristics of CMMCs and their electrical correlates are, at least in adult mice, indistinguishable from wild type control offspring. One possibility why the major characteristics of CMMCs were no different between both types of mice is that nNOS KO mice may compensate for their life long deletion of the nNOS gene, and their permanent loss of neuronal NO production. In this regard, we suggest caution should be exercised when assuming that data obtained from adult nNOS KO mice can be directly extrapolated to wild type mice, that have been acutely exposed to an inhibitor of NOS.

## Introduction

Colonic migrating motor complexes (CMMCs) are one of the major types of motor pattern that occur in the large intestine of a variety of mammals both *in vivo* (Ferre and Ruckebusch, 1985; Sarna, 1991; Morita et al., 2012) and *in vitro* (Wood, 1973; Fida et al., 1997; Bywater et al., 1998; Brierley et al., 2001; Powell and Bywater, 2001; Roberts et al., 2007, 2008; Heredia et al., 2009; Copel et al., 2013). CMMCs have also been reported in human colon *in vivo* (Hagger et al., 2003) and recently in the intact whole isolated human colon (Spencer et al., 2012). Although the function of CMMCs is not entirely clear, it is likely they underlie, at least in part, propulsion of semi solid and solid content along the large bowel.

In mouse colon, the mechanisms that underlie intracellular electrophysiological properties of CMMCs have been studied in detail by a number of investigators over the past 20 years (Bywater et al., 1989, 1998; Lyster et al., 1995; Spencer et al., 1998b, 2005; Dickson et al., 2010). Unlike electrical slow waves which are myogenic in origin (Huizinga et al., 1995) and do not require the presence of enteric nerves for their generation (Ward et al., 1999), CMMCs are critically dependent upon the enteric nervous system, since they are abolished by hexamethonium or tetrodotoxin (Wood, 1973; Bywater et al., 1989; Lyster et al., 1995; Fida et al., 1997; Spencer et al., 1998b). And, CMMCs do not occur in regions of colon that are aganglionic, lacking enteric nerves (Spencer et al., 2007). Whilst slow waves have been recorded from the mouse colon (Wood, 1973; Lyster et al., 1993), they are rare, and occur at vastly different frequencies (16 per min, Wood, 1973; Lyster et al., 1993), compared with electrically recorded myoelectric complexes (MCs) (that underlie CMMCs), occurring every 2–5 min; see (Bywater et al., 1998), for review. Also, whilst interstitial cells are essential for slow wave generation (Huizinga et al., 1995), studies have shown that in *W/W*^*v*^ mutant mice, that lack intestinal slow waves and pacemaker type ICC-MY, cyclical MMCs were still reliably and consistently recorded, which were abolished by hexamethonium or atropine (Spencer et al., 2003). A recent electrophysiological study of mouse GI-tract indeed confirmed that whilst slow waves were regularly recorded from the upper GI-tract, they were not recorded from mouse cecum (Gil et al., 2013).

The intrinsic neural mechanisms that underlie the generation of CMMCs involves activation of complex enteric neural pathways (Bywater et al., 1989, 1998; Lyster et al., 1995; Spencer et al., 1998b, 2005; Dickson et al., 2010). CMMC generation involves two major processes: (1), activation of cholinergic motor neurons causing cholinergic (atropine-sensitive) oscillations in membrane potential (occurring at 2 Hz) (Bywater et al., 1989, 1998; Lyster et al., 1993, 1995; Spencer et al., 2005; Dickson et al., 2010) and; (2) inhibition of ongoing inhibitory neurotransmitter release (disinhibition) (Lyster et al., 1995; Spencer et al., 1998b). Indeed, the process of disinhibition was first described for the periodic generation of migrating spike bursts along the isolated cat colon (Christensen et al., 1978). In the mouse colon, the process of disinhibition was shown to involve the suppression of both nitrergic and non-nitrergic neurotransmitters, acting via presynaptic suppression of inhibitory transmitter output (Spencer et al., 1998a). Although disinhibition involved, in part, the periodic suppression of nitric oxide (NO) release, CMMCs and the disinhibition process itself still occurred when endogenous NO production was blocked. This was demonstrated when it was found that CMMCs still occurred in the presence of NOS inhibitors and in fact, CMMCs occurred more frequently (Fida et al., 1997; Powell and Bywater, 2001). Also, inhibitors of NOS were found to significantly depolarize the resting membrane potential (RMP) of the circular muscle, by decreasing the repolarization phase during the intervals between MCs (Lyster et al., 1995; Spencer et al., 1998a,b). This showed that whilst the identity of the pacemaker cell(s) that generate CMMCs remains unclear, the timing and frequency of CMMCs is strongly modulated by ongoing neuronal release of NO (Fida et al., 1997; Spencer et al., 1998a, b; Spencer, 2001; Roberts et al., 2007), in addition to maintaining tonic inhibition of the circular muscle between MCs (Spencer et al., 1998a, b). In further support of this, a recent study showed that CMMCs still occur in neuronal nitric oxide synthase (nNOS) knockout (KO) mice (Dickson et al., 2010), and it was reported that they occurred more frequently compared to wild type littermates (Dickson et al., 2010). In this study, we have recorded the electrical and mechanical characteristics of nNOS KO mice to determine whether permanent loss of nNOS leads to similar CMMC characteristics as in wild type mice that have endogenous NO synthesis prevented acutely, by pharmacological blockers of NOS.

## Methods

### Preparation of tissues

Neuronal nitric oxide synthase (nNOS) KO mice (30–90 days of age) of either sex and their wild type control littermates were obtained from the Jackson Laboratory, Maine, U.S.A. Mice were euthanized humanely by isoflurane inhalation overdose, followed by cervical dislocation, as approved by the local animal welfare committee. The entire colon was removed from mice and placed in room temperature Krebs solution, which was constantly bubbled with carbogen gas (95% O2/5% CO2).

### Mechanical recordings from the circular muscle during spontaneous CMMCs

We recorded the force generated by the circular muscle layer during each spontaneous CMMC, using an isometric recording transducer (Grass FT-03C; Grass, Quincy, M.A., U.S.A) connected via fine suture thread to hooks that pierced the muscle wall. Mechanical recordings were made under isometric conditions, using force transducers that were connected to two custom made preamplifiers (Biomedical engineering, Flinders University) and then to a Powerlab (model: 4/30; AD Instruments, Bella Vista, N.S.W, Australia). Labchart version 6.0 (AD Instruments, Australia) was used for analysis of data.

### Intracellular electrophysiological recordings from circular muscle cells

Intracellular recordings were made from circular muscle cells in the mid-distal region of isolated whole mouse colon, using an independently mounted micromanipulator (model M3301R; WPI Inc. Sarasota, FL. USA). All recordings were made in the presence of atropine (1 μM) and nifedipine (1 μM). Microelectrodes (i.d. 0.5 mm) were filled with 1.5 M KCl solution and having tip resistances of about 100 MΩ. Electrical signals were amplified using a dual input Axoprobe 1A amplifier and digitized at between 660 Hz and 1.5 KHz on a PC using Axoscope software (version 8.0; Axon Instruments, Foster City, CA, USA).

### Measurements and statistics

Measurements of the peak amplitude and interval between CMMCs and MCs were measured from isometric mechanical recordings using Chart Version 6.0 (AD Instruments, N.S.W. Australia) and intracellular recordings. Data in the results section are presented as means ± S.E.M. The use of “n” in the results section refers to the number of animals on which observations were made. Data sets were considered statistically significant if *P* values < 0.05 were reached. Statistical analysis of data was conducted for all experiments using within-fields/repeated measures analysis.

### Drugs and solutions

The Krebs solution used contained (in mM): NaCl, 118; KCl, 4.7; NaHPO4.2H20, 1.0; NaHCO3, 25; MgCl.6H20, 1.2; D- Glucose, 11; CaCl2.2H20, 2.5. L-NA was obtained from Sigma Chemical Co. Mo. U.S.A and was made up as a stock solution of 10 mM in deionized water.

## Results

### Observations from mechanical recordings of CMMC activity in wild type and nNOS KO mice

**Figure 1:**
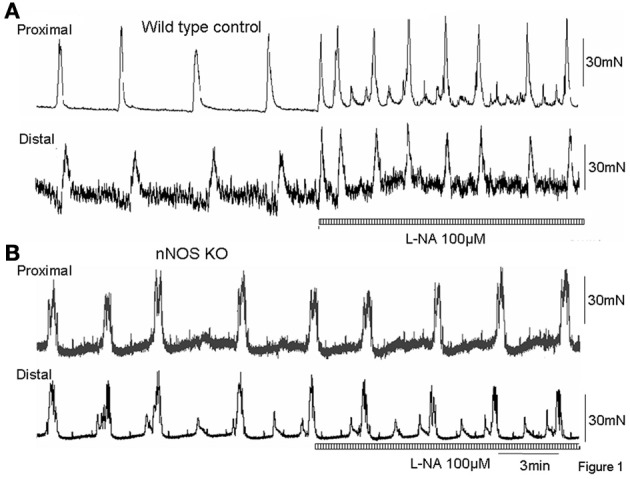
**(A)** Mechanical recordings from the circular muscle layer from proximal and distal colon of a wild type control mouse. CMMCs occur approximately every 3–4 min. Addition of the NO inhibitor, L-NA (100 μM) caused a significant increase in frequency of CMMCs. **(B)** in an nNOS KO mouse, surprisingly, CMMCs occur at a similar frequency as wild type control animals (as in **A**), but addition of the NOS inhibitor, L-NA has no effect confirming this is a KO mouse.

**Figure 2:**
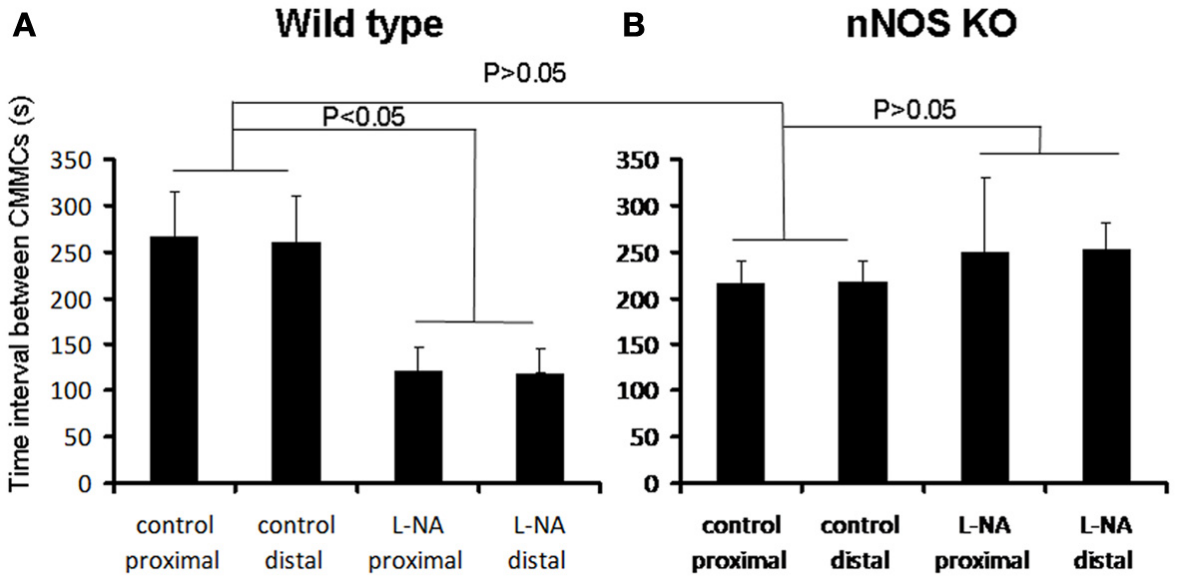
**Graphical representation of the changes in CMMC interval in wild type and nNOS KO mice (A) in wild type control mice, L-NA caused a significant reduction in the interval between CMMCs. (B)** in nNOS KO mice the mean interval between CMMCs was not different from the mean interval between CMMCs in control mice. Addition of L-NA had no effect on CMMC intervals in nNOS KO mice.

Mechanical recordings were made from the circular muscle of proximal and distal colon of isolated whole mouse colon in wild type control mice (*N* = 8). During these recordings, CMMCs were found to occur every 260.7 ± 40 s (Figure 1A; *N* = 8). When the NOS inhibitor, L-NA (100 μM) was applied to the colon it significantly increased the frequency of occurrence of CMMCs, such that their mean interval decreased to 121.7 ± 25 s (Figure 2; *P* < 0.05; Figure 1A; *N* = 8).

We anticipated that the frequency of CMMCs in nNOS KO mice would be significantly faster than in control mice, and mimic the frequency of CMMCs in wild type mice when exposed to an inhibitor of NOS. We found that the mean interval between CMMCs in nNOS KO mice was 218.7 ± 25 s, which was no different from the control frequency of CMMCs in wild type mice (Figure 2; *P* > 0.05; *N* = 8). Addition of L-NA (100 μM) to nNOS KO mice, had no effect on CMMC frequency (Figure 1B; *P* > 0.05; *N* = 8).

### Recordings of intracellular electrical activity from the circular muscle cells during MCS in nNOS KO and wild type mice

**Figure 3:**
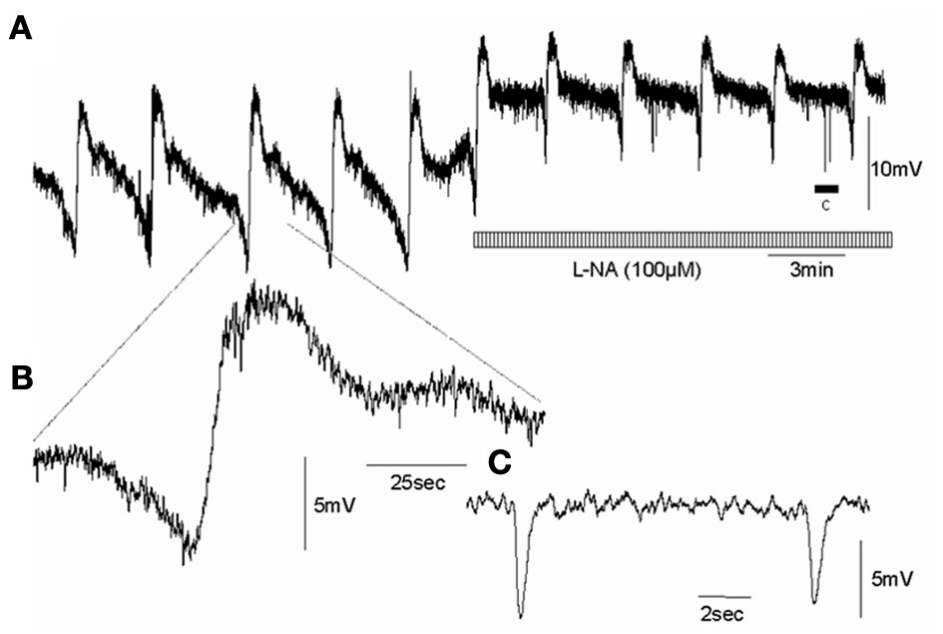
**Effects of an inhibitor of NOS on intracellularly recorded MCs in the circular muscle layer of wild type control mouse whole colon, whilst in the presence of nifedipine (A) CMMCs occur with a pre-complex hyperpolarization and slow membrane depolarization.** Addition of L-NA induced significant depolarization and increased the frequency of MCs. **(B)** shows a single myoelectric complex. **(C)** shows the period of spontaneous inhibitory junction potentials represented by the arrow c, in panel **(A)**. Note, the spontaneous fast IJPs are unaffected by NOS blockade.

**Figure 4:**
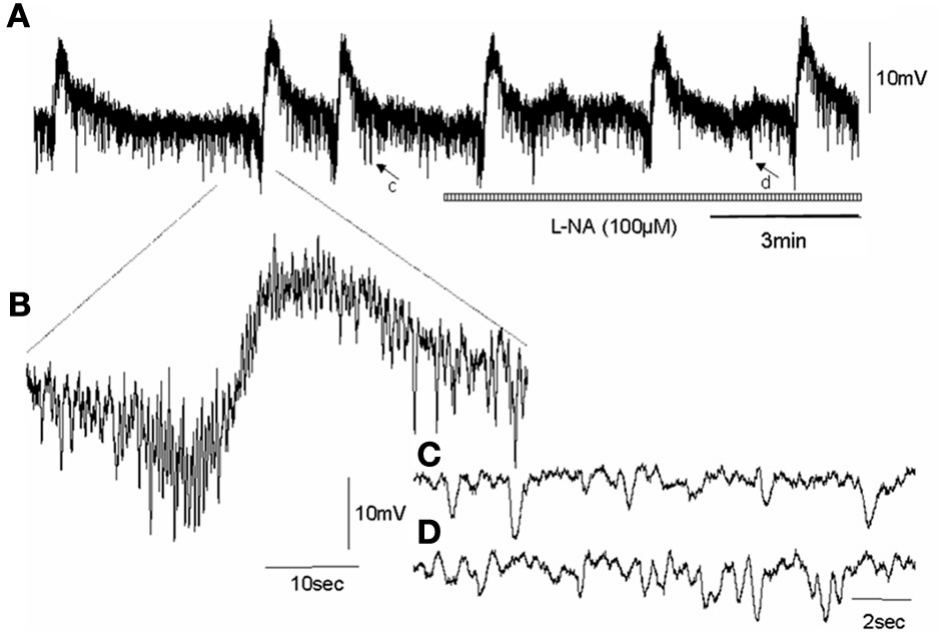
**Shows effect of NOS inhibition on an intracellular recording of MCs in the circular muscle layer of an nNOS KO mouse colon, in the presence of nifedipine. (A)** CMMCs occur with a pre-complex hyperpolarization and slow membrane depolarization in nNOS KO mice, similar to wild type controls. Unlike wild type animals, addition of L-NA did not induce any depolarization of the circular muscle layer and did not increase the frequency of MCs. **(B)** shows a single myoelectric complex. **(C)** shows a brief recording represented by the arrow c in panel **(A)** of spontaneous IJPs that occurs in Krebs solution prior the addition of L-NA. **(D)** shows the period of recording represented by the arrow in d in panel **(A)**, of spontaneous inhibitory junction potentials in the presence of L-NA. There is no change, as expected, in the fast IJPs recorded in nNOS KO mice.

In wild type control mice, the RMP of the circular muscle layer during the intervals between MCs was −43.7 ± 1.2 mV (26 cells, *n* = 11) and during the rising phase of MCs, the mean peak amplitude of slow depolarization was 12.6 ± 1.4 mV (15 cells, *n* = 11). Addition of L-NA (100 μM) cause a significant depolarization of the circular muscle and reduced the amplitude of MCs (*P* < 0.05; Figure 3; *N* = 11). Surprisingly, in nNOS KO mice the RMP was no different from wild type animals at −42.4 ± 0.9 mV (61 cells, *P* > 0.05; *n* = 15). Also, the amplitude of slow depolarization phase during MCs was 11.2 ± 1.4 mV, which was also no different from controls (14 cells, Figure 4; *P* > 0.05; *n* = 9).

### Confirmation that nNOS KO mice lack neuronal nitric oxide

**Figure 5:**
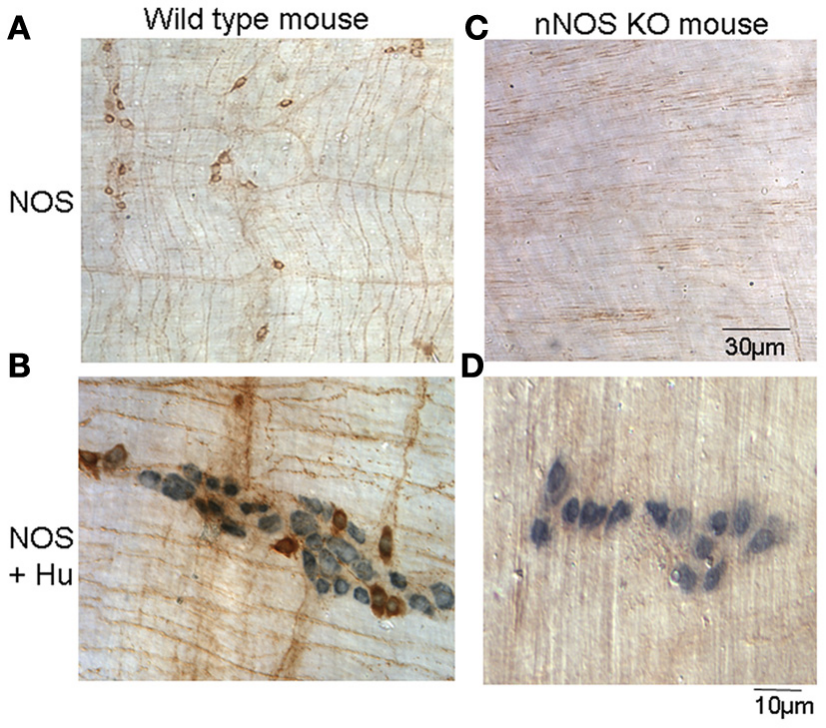
**Immunohistochemical confirmation that nNOS KO mice lack nNOS in enteric neurons of mouse colon. (A)** shows nNOS positive cell bodies and fibers shown in brown in myenteric ganglia and smooth muscle, respectively. **(B)** shows colocalization of nNOS positive neuronal cell bodies (brown) and all enteric neurons labeled by the general neuronal marker, Hu (see blue cell bodies). **(C)** shows an nNOS KO mouse has a lack of enteric neurons that are NOS positive and fibers in the circular muscle. **(D)** shows there is no co-localization of nNOS and Hu cell bodies. Only Hu positive cell bodies are present in myenteric ganglia of nNOS KO mice.

We used a neuronal NOS antibody to confirm the presence or absence of neuronal NOS in wild type and nNOS KO offspring. In wild type offspring, nNOS positive neurons were always present in myenteric ganglia (Figure 5A). Also, the general neuronal antibody, Hu, was used to confirm that nNOS positive neurons were Hu positive. This was always the case (Figure 5A). In nNOS KO mice, no nNOS immunoreactive neurons were detected (*N* = 5; Figure 5B). To confirm that enteric neurons were present in nNOS KO mice, we also used the Hu antibody and found that despite the absence of nNOS positive neurons (Figures 5C,D), Hu positive enteric neurons were present (Figure 5B; *N* = 5).

## Discussion

It is well known that endogenous NO release is not required for the generation of CMMCs or their underlying myoelectrical correlates in mouse colon, *in vitro*. However, endogenous NO potently modulates the frequency of CMMCs and maintains the RMP of the CM in a hyperpolarized state, during the intervals between CMMCs. The major aim of the current study was to determine whether nNOS KO mice would show CMMC characteristics that are similar to CMMCs recorded from wild type mice, when acutely exposed to an inhibitor of NOS. The major finding in this study is our unexpected observation that the frequency of CMMCs in nNOS KO mice was not significantly different from wild type control mice. Also, unexpectedly, the RMP of the circular muscle layer was not significantly more depolarized compared to control mice and the amplitude of the slow membrane depolarization underlying MCs was not different from control.

### Why are CMMCs in nNOS KO mice not different from controls?

We expected that CMMCs would occur more frequently in nNOS KO mice and MCs underlying CMMCs to be of reduced amplitude. The reason why we expected these results was because we had found previously that acute blockade of NOS caused these effects in wild type mice where nNOS is naturally expressed. However, when we studied nNOS KO mice, none of our expectations were supported by our data. The fact that in nNOS KO mice there was no overall difference in the major characteristics of CMMCs, or their underlying electrical correlates, suggests substantial compensation occurs throughout the life of these animals. This could occur via upregulation of other neurotransmitters/receptors, ion channels, or signaling pathways. Although our study did not reveal differences in the frequency of CMMCs between nNOS KO mice and wild type offspring, another study characterized the properties of CMMCs in nNOS KO mice and concluded that CMMCs did occur significantly more frequently in nNOS KO mice, than in wild type controls (Dickson et al., 2010). We are unable to explain these differences. Interestingly, the records presented in Dickson et al. (2010) (see Figure 2B) do not show any clear difference in CMMC frequency between nNOS KO mice and wild type siblings.

### NOS inhibitors have no effect on CMMCs or MC motor patterns in nNOS KO mice

Whilst acute exposure of an NOS inhibitor in wild type mice caused an immediate and pronounced increase in CMMC and MC frequency, application of an NOS inhibitor to nNOS KO mice did not cause any detectable changes in CMMCs or MCs (Figures 3, 4). This is an important observation, because it suggests that nNOS is specifically responsible for modulating CMMC and MC frequency; and that iNOS and eNOS (both present in the mouse colon), do not play a detectable role in modulating CMMC characteristics, at least *in vitro*.

### How do these findings in nNOS KO mice relate to previous studies

Based on our previous studies (Spencer et al., 1998a, b), we assumed that mice with a genetic deletion of the nNOS gene would show depolarized RMPs during the intervals between MCs. In fact, we did not find any difference between the RMPs in wild type control mice compared with nNOS KO mice. Indeed, when an NOS antagonist was applied to wild type mouse colon, the RMP between MCs depolarized significantly, consistent with previous findings (Spencer et al., 1998a, b). In contrast, addition of an NOS inhibitor to nNOS KO mice had no effect on RMP of the circular layer, nor, on the mechanical activity underlying CMMCs. This result in nNOS KO mice suggests that during the intervals between CMMCs, the RMP of the circular muscle layer is maintained in a hyperpolarized state by neurotransmitters other than NO and/or upregulation of other receptors, signaling pathways or second messenger systems. The nature of which requires further investigation. Although we could not demonstrate depolarized RMPs in nNOS KO mice, a recent study by Dickson et al. (2010) reported that the RMP of the circular muscle was significantly depolarized compared to wild type mice.

## Conclusions

The findings of the current study show that the CMMC motor pattern recorded from nNOS KO mice (that lack neuronal NO throughout their entire life), do not provide comparable characteristics to wild type mice exposed to an NOS inhibitor. We suggest caution should be exercised when assuming, or expecting, that the motility patterns recording from nNOS KO mice can be directly extrapolated to, and compared with, the data obtained from wild type mice that have been acutely exposed to an inhibitor of NOS.

### Conflict of interest statement

The author declares that the research was conducted in the absence of any commercial or financial relationships that could be construed as a potential conflict of interest.
